# Green Synthesis of Copper Nanoparticles Using *Sargassum* spp. for Electrochemical Reduction of CO_2_

**DOI:** 10.1002/open.202300190

**Published:** 2024-01-09

**Authors:** Sandra Jazmín Figueroa Ramírez, Beatriz Escobar Morales, Diego Alonso Pantoja Velueta, Juan Manuel T. Sierra Grajeda, Ivonne Liliana Alonso Lemus, Claudia Alejandra Aguilar Ucán

**Affiliations:** ^1^ Facultad de Ingeniería Universidad Autónoma del Carmen Av. Central S/N, Esq. con Fracc. Mundo Maya Ciudad del Carmen 24115 Campeche, México; ^2^ CONAHCYT – Centro de Investigación Científica de Yucatán 5.5 Carretera Sierra Papacal-Chuburná Puerto Sierra Papacal Yucatán 97302, México; ^3^ CONAHCYT – Cinvestav Unidad Saltillo Sustentabilidad de los Recursos Naturales y Energía Av. Industria Metalúrgica, Parque Industrial Saltillo-Ramos Arizpe Coah Ramos Arizpe 25900 México; ^4^ Facultad de Química Universidad Autónoma del Carmen Calle 56, No. 4 Av. Concordia Ciudad de Carmen Campeche 24180, México.

**Keywords:** copper nanoparticles, *Sargassum* spp., biocarbon, CO_2_ reduction

## Abstract

This study presents a green method of producing copper nanoparticles (CuNPs) using aqueous extracts from *Sargassum* spp. as reducing, stabilizing, and capping agents. The CuNPs created using this algae‐based method are not hazardous, they are eco‐friendly, and less toxic than their chemically synthesized counterparts. The XRD characterization of the CuNPs revealed the presence of Cu and CuO, with a crystallite size ranging from 13 to 17 nm. Following this, the CuNPs were supported onto a carbon substrate, also derived from *Sargassum* spp. (biochar CSKPH). The CuNPs in biochar (CuNPs‐CSKPH) did not appear in the XRD diffractograms, but the SEM‐EDS results showed that they accounted for 36 % of the copper weight. The voltamperometric study of CuNps‐CSKPH in acid media validated the presence of Cu and the amount was determined to be 2.58 μg. The catalytic activity of CuNPs‐CSKPH was analyzed for the electrochemical reduction of CO_2_. The use of *Sargassum* spp. has great potential to tackle two environmental problems simultaneously, by using it as raw material for the synthesis of activated biochar as support, as well as the synthesis of CuNPs, and secondly, by using it as a sustainable material for the electrochemical conversion of CO_2_.

## Graphical abstract

CuNPs were synthesized using a cost‐effective and environmentally friendly extract applying *Sargassum* spp. as versatile reducing agent. In this study, CuNPs with nanoflower morphology were synthesized. The experimental characterizations (XRD, SEM, and TEM,) recommend that the green synthesis method has promising effect for the synthesis of CuNPs, stable and homogeneously dispersed onto biochar. The CuNPs‐CSKPH is promising electrocatalyst for the electrochemical reduction of CO_2_.

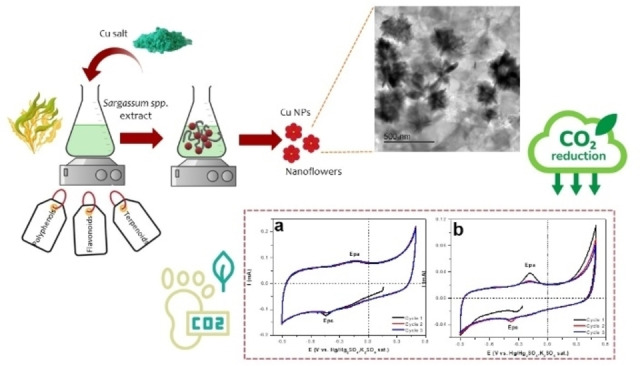

## Introduction

Nowadays, several physical, chemical, biological, and hybrid processes are available for synthesizing nanoparticles (NPs), each with unique characteristics. However, there is a growing need to develop synthesis processes that prioritize green chemistry, cost‐effective, eco‐friendly, safe to handle, simple, reproducible, easily scalable for higher production, and less energy expenditure requirement.^[1]^ The use of plant extracts is considered an environmentally friendly method. It plays an essential role in its synergy with green chemistry because the reduction, stabilization, and type of solvent determine the morphological characteristics (spheres, rods, prisms, plates, needles, leaves) and the size of the NPs.

Algae are an abundant and widely available natural resource, and they are considered a natural repository of minerals, vitamins, proteins, and other bioactive substances, which act as reducing agents in the synthesis of metallic NPs.^[2]^ *Sargassum* spp. is a macro‐algae belonging to the genus Phaeophyceae of the *Sargassaceae* family that includes around 400 species; it is a brown alga whose average composition by weight is 14.33 % moisture, 6.55 % protein, 1.90 % lipid, 18.50 % ash, 58.72 % carbohydrates, and 17.00 % fiber. Studies reveal that there are about 200 bioactive components present in *Sargassum spp*.^[3]^ The synthesis of CuNPs using extracts from *Sargassum* spp. would provide many benefits caused by its various applications. In addition, seaweeds are not widely used for human consumption, adding a very long‐term advantage because significant fluctuations in their use are not expected as a result of overpopulation.^[4]^ An additional advantage is that sargassum is currently considered a serious environmental problem that adds to the problem of CO_2_ emissions from anthropogenic sources. This is why its use as a stabilizing, reducing, and capping agent in the synthesis of copper nanoparticles (CuNPs) represents an option viable in the short term.

**Figure 1:**
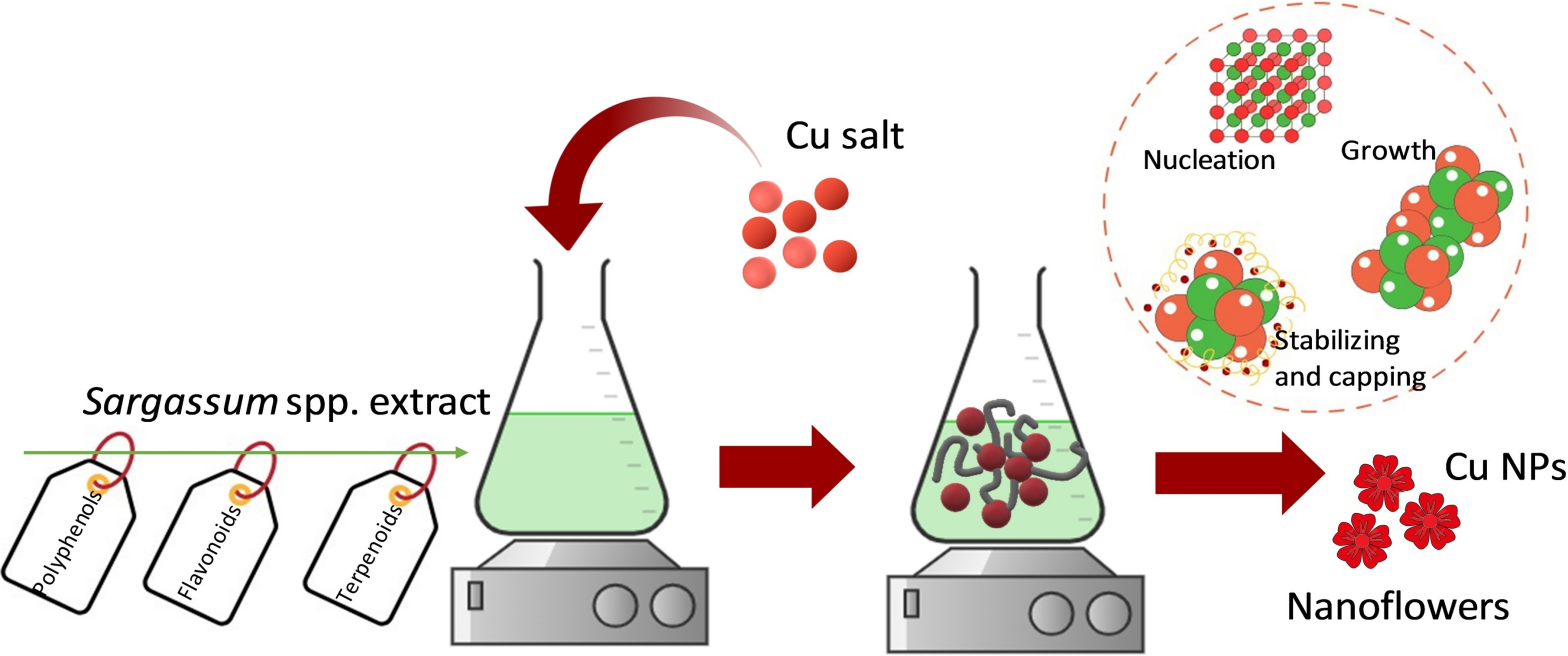
Methodology utilized for the green synthesis of CuNPs.

CuNPs are considered one of the cheapest transition metals. The interest in their study is attributable to their excellent chemical, physical, optical, catalytic, large surface area to volume ratio, stability, magnetic, and biological properties,^[5]^ making them useful in a wide range of applications. Essential parameters for excellent performance are related to the sizes, shapes, compositions, and structures of the nanoparticles. The synthesis of CuNPs begins with a redox reaction by mixing the extract of *Sargassum* spp. with copper salt; this process comprises the reduction of copper ions in the presence of active ingredients, contributes to the formation of Cu^0^ and then, through agglomeration, forms oligomeric clusters, thus leading to colloidal CuNPs. It is considered a reaction between metabolites and chemical groups found in the cell wall of dead biomass or extracellular metabolites, in addition to being a spontaneous reaction,^[6]^ Figure 1. Some authors have reported the synthesis of CuNPs using various plant extracts using every part as the stem, flower, fruit, and root.[^7^, ^8^, ^9^] Recently, the CuNPs have been used for the CO_2_ reduction reaction (CO_2_RR) because of their excellent capacity to reduce CO_2_ to many products like methanol, ethanol, propanol, and other products with different added values^[10]^ by changing parameters such as the applied potential and electrolyte. In addition, ECR meets other competitive technological advantages, among which is that it generally requires ambient temperature and pressure and can incorporate renewable energy sources in the processes.^[11]^ Dongare *et al*.^[12]^ prepared CuNPs, which showed porous morphology in a pure metallic state with a high surface area of 630 m^2^ g^−1^. The synthesized nanoparticles show electrocatalytic reduction activity towards formate/formic acid, acetate, ethanol, and n‐propanol formation after coating on the carbon paper. Xiao *et al*.^[13]^ synthesized copper nanoparticles dispersed carbon aerogels with a high surface area of 867.87 m^2^ g^−1^. These nanoparticles enhanced the electrochemical reduction of CO_2_ in 0.1 M KHCO_3_ aqueous media.

It is worth mentioning that to perform the electrochemical CO_2_ reduction efficiently, the use of suitable supports is required. Traditionally carbon‐based materials are employed because of their low cost, high surface area, mesoporous and microporous structure, and excellent electrical conductivity. Hence, a combination of CuNPs and biochar was obtained from *Sargassum* spp. provide an important field of opportunity effectively to improve its application in the ECR.

## Results and Discussion

### Characterization of SCuNPs by XRD

**Figure 2:**
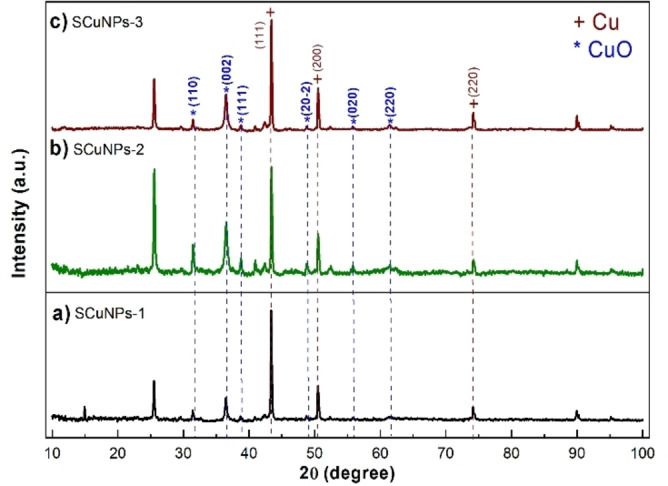
X‐ray diffraction (XRD) patterns of copper nanoparticles (a) SCuNPs‐1, (b) SCuNPs‐2, and (c) SCuNPs‐3 obtained by green synthesis.

Figure 2 shows the XRD analysis of synthesized CuNPs with different ratios of extract SE and the copper precursor obtained by green synthesis. It confirms the formation of copper with mixed oxidation states. In all samples, the presence of significant diffraction peaks of the fcc structure of pure Cu (JCPDS No. 71.4610) is observed at 43.3°, 50.5° and 74.1° corresponding to the (111), (200), and (220) planes.^[14]^ In addition, other diffraction peaks of CuO appear (JCPDS No. 48–1548) that confirm the formation of oxides.^[15]^

The crystallite size was determined from the full width at half maximum intensity (FWHM) by the maximum peak of the diffraction plane (111) of copper with a face‐centered cubic structure and by applying the Scherrer equation.^[16]^ Table 1 summarizes the obtained values for the three samples showing similar crystallite sizes.

**Table 1:** Crystallite size of different SCuNPs.

Copper nanoparticles	Crystallite size (nm)
SCuNPs‐1	13.27
SCuNPs‐2	15.13
SCuNPs‐3	17.21

### Characterization of SCuNPs by TEM

**Figure 3:**
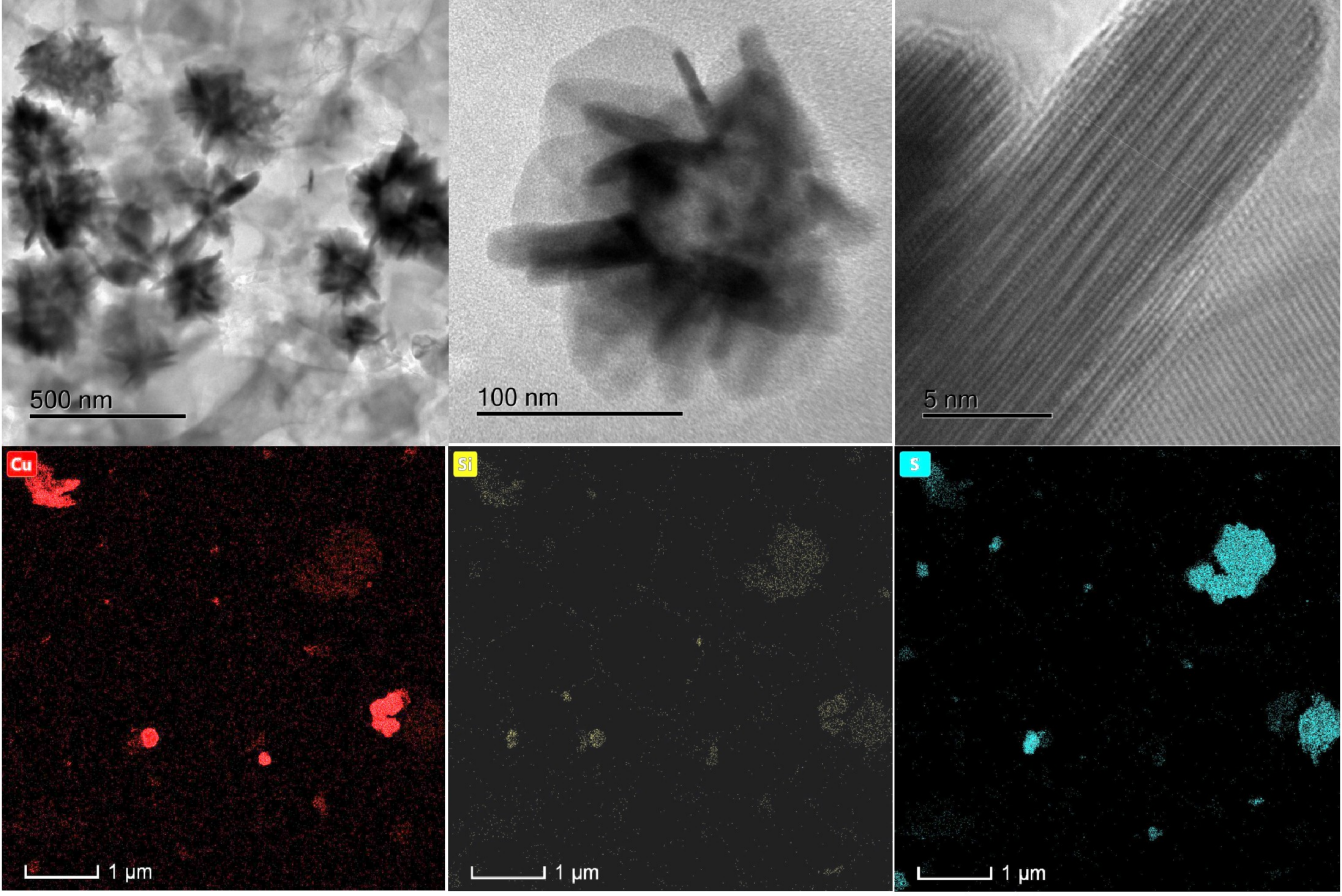
(a–c) Representative TEM images of CuO and Cu nanoflowers by green synthesis and (d–f) elemental mapping images.

The TEM experiments showed that the morphologies of the SCuNPs‐3, Figure 3 (a–c), correspond to nanoflakes formed by the accumulation of the CuO and Cu nuclei, which aggregate to form flower‐like particles that grow in different directions with almost uniform sizes.[^17^, ^18^, ^19^] Besides, CuO and Cu nanoflakes of different sizes comprise aggregating nanorods. The width of the flakes was estimated to be 8.3 nm (Figure 3c). The CuO and Cu formation processes start with nucleation, followed by aggregation and self‐assembly to form larger particles. The interplanar distance of CuO was 2.3 Å. The elemental mapping of SCuNPs‐3 shows an agglomeration, Figure 3 (d–f), as in the TEM analysis; Cu, Si, and S elements are present over nanoflowers.

### Characterization of the Catalysts Through XRD

**Figure 4:**
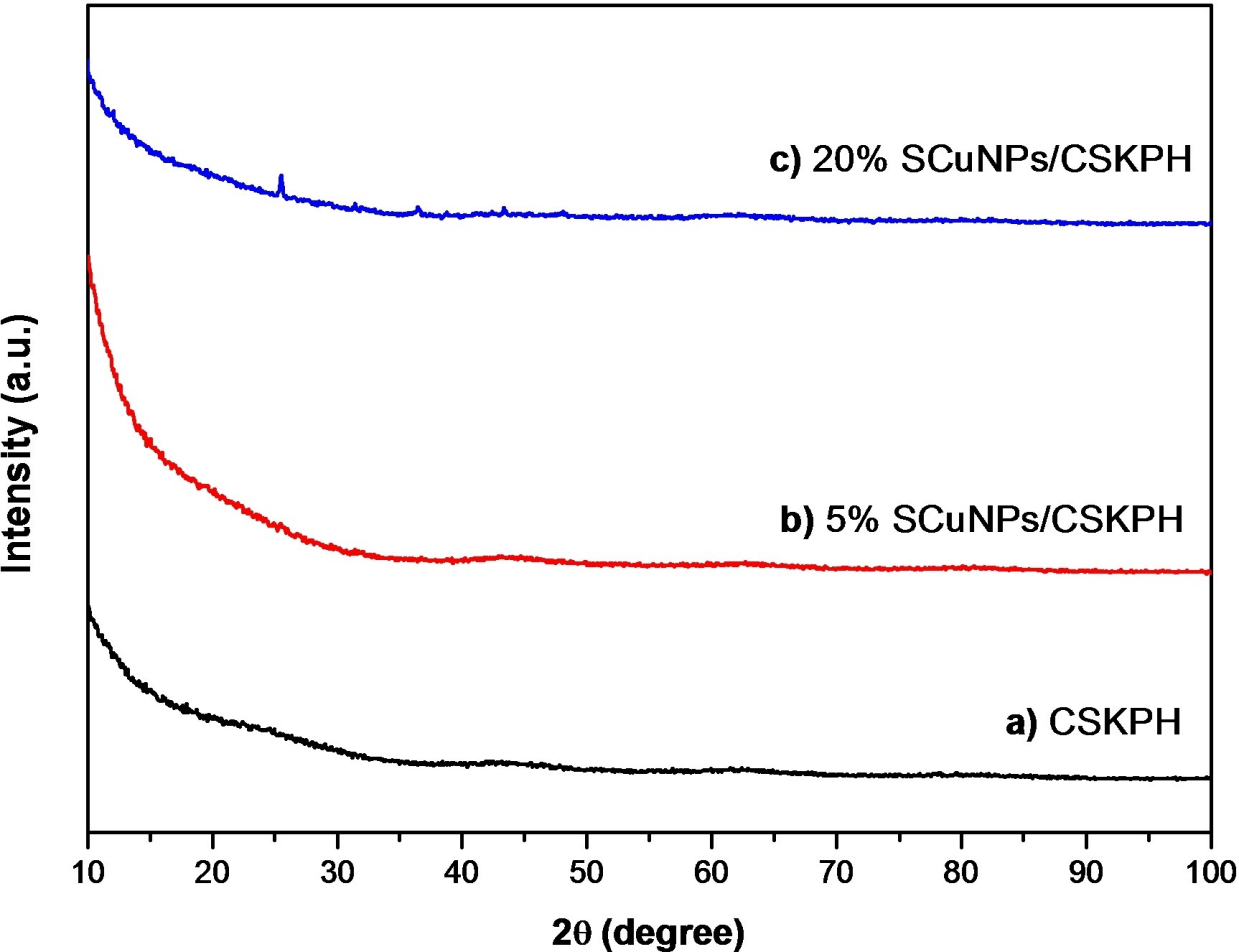
X‐ray diffraction (XRD) patterns of (a) CSKPH, (b) 5 % SCuNPs/CSKPH, and (c) 20 % SCuNPs/CSKPH synthesized by green synthesis.

Figure 4 shows diffractograms corresponding to biochar (CSKPH) and the catalysts 5 % SCuNPs/CSKPH and 20 % SCuNPs/CSKPH. No reflection is associated with the amorphous nature of CSKPH biochar observed.^[20]^ Neither are characteristic reflection peaks observed from Cu species that can be detected, which may be owing to the low content of Cu.

### Morphological and Chemical Characterization of the Catalyst by SEM‐EDS

**Figure 5:**
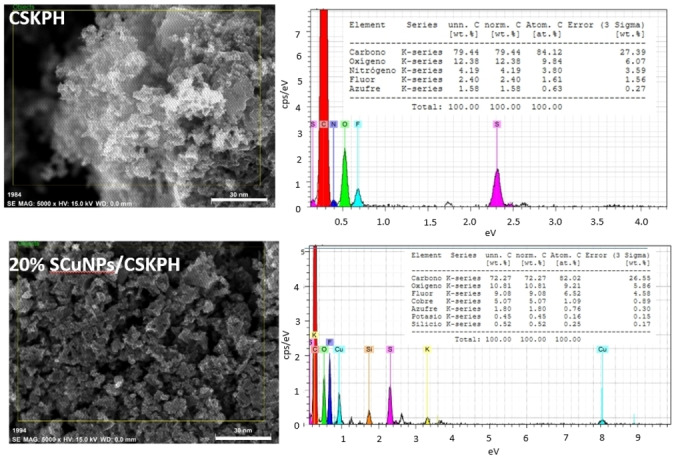
SEM‐EDS Images of the CSKPH and 20 % SCuNPs/CSKPH.

Figure 5 shows the SEM‐EDS micrograph of the CSKPH biochar, and the 20 % SCuNPs/CSKPH catalyst obtained by green synthesis. In the CSKPH sample, a porous and homogeneous surface with 36 % carbon is observed, as well as the presence of O, Si, S, Cl, and Fe is assignable to the synthesis process that includes KOH, while Si is associated with the source of biomass. In the 20 % SCuNPs/CSKPH catalyst, agglomerations result from alkaline residues on the catalyst surface. This analysis shows 5 % of the copper mass and 39 % carbon; the remaining elements are associated with the catalyst synthesis process.

### Cyclic Voltammetry Study of Catalyst

**Figure 6:**
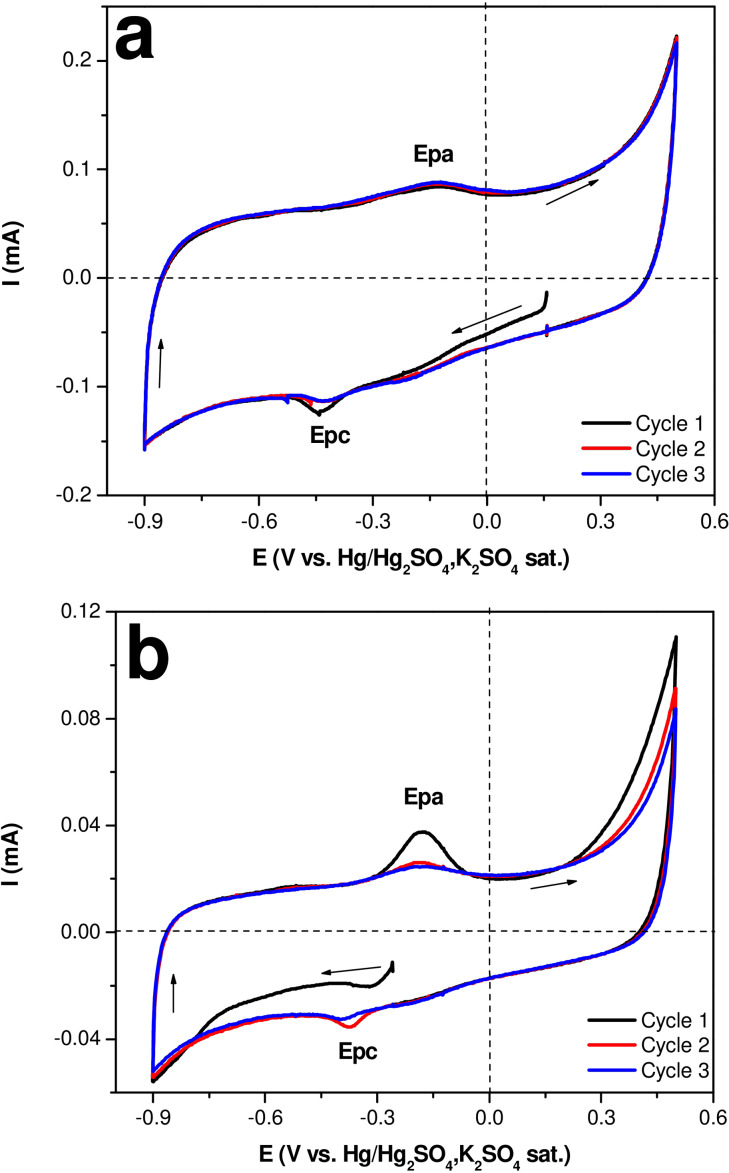
Voltammetric responses were obtained on a) CSKPH film and b) 20 % SCuNPs/CSKPH in the 0.1 M K_2_SO_4_ system with pH=2.3 at 5 mV s^−1.^

Through SEM‐EDS, it was possible to know the copper percentage; however, to quantify the amount of copper present in the 20 % SCuNPs/CSKPH catalyst, a cyclic voltammetry (CV) study was performed. Figure 6 shows the voltammetric responses obtained on the CSKPH and 20 % SCuNPs/CSKPH films in the 0.1 M K_2_SO_4_ system at pH=2.3 to 5 mVs^−1^ for three cycles. First, the potential scanning was started in a negative direction regarding open circuit potential (E_ocp_). Figure 6 (a) depicts the behavior of CSKPH film, which exhibits a pseudocapacitive behavior as a consequence of charge accumulation and a redox process associated with a functional group on the surface of biochar. The broad peak between −0.3 V and 0.2 V can be related to the C−OH groups reported in^[21]^ or the quinone/hydroquinone pair. Figure 6 (b) shows the response of 20 % SCuNPs/CSKPH. It reveals a small peak of −0.31 V, which is associated with the reduction of the carbon functional group (first cycle, black line). On inverting the potential scan, an anodic peak potential (Epa) of −0.18 V is observed, which is linked to the oxidation of SCuNPs. As the cycles are performed, the cathodic peak current (Ipc) increases negatively on account of the reduction of the copper oxidized in the first cycle (equation 1). Subsequently, the cathodic peak potential (Epc) at −0.37 V associated with the reduction of copper dissolved in the first cycle is described. The voltammogram shows that the Ipa decreases as the cycles are carried out, indicating the dissolution of the copper contained in the catalyst.

(1) $$Cu\vec{\leftarrow }{Cu}^{2+}+2e^{-}$$

Table 2 shows the parameters of the potential cathodic and anodic peak potential; and the anodic peak current of the first cycle. Comparing the results, the values of cathodic and anodic peak potential were very similar; however, the values of Ipa of catalyst of 20 % SCuNPs/CSKPH decrease as the cycle number increases; this observation might be related to the copper dissolution that is present on the porous surface of CSKPH.

**Table 2:** Parameters of cathodic and anodic peak potentials and the anodic current obtained from the voltammograms of CSKPH and 20 % SCuNPs/CSKPH.

Electrodes	1st cycle		1st cycle	2nd cycle	3rd cycle
	Epc (V)	Epa (V)	Ipa (mA)		
CSKPH	−0.4412	−0.1345	0.0841	0.0867	0.0877
20 % SCuNPs/ CSKPH	−0.3734	−0.1815	0.0375	0.0260	0.0245

The first Faraday law determined the amount of copper in the catalyst (Table 3). In the first cycle, 2.28 μg of copper was obtained; as the number of cycles increased, the mass decreased; therefore, the total amount of copper contained in the catalyst was 6.29 μg.

**Table 3:** Copper charge and mass values are obtained in each cycle.

Cycle	Q×10^3^ (C s^−1^)	m (μg)
1	6.93	2.28
2	6.17	2.03
3	6.00	1.98

This study allowed us to determine the amount of copper in the catalyst, 20 % SCuNPs/CSKPH.

### Evaluation of 20 % SCuNPs/CSKPH Electrochemically Active Surface Area (EASA)

**Figure 7:**
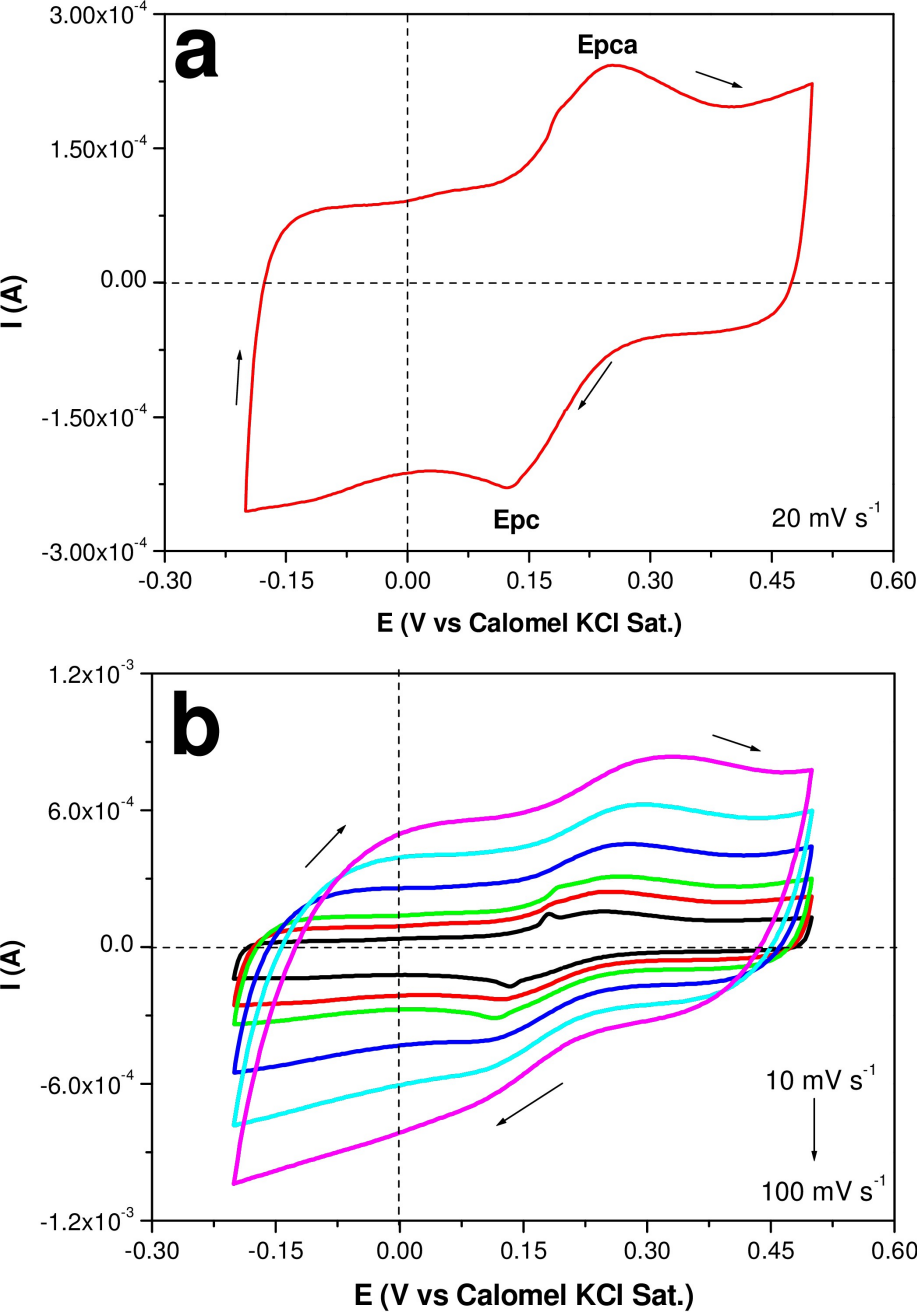
CV curves SCuNPS/CSKPH in 0.005 M K_3_Fe(CN)_6_, 0.005 M K_4_Fe(CN)_6_, 0.1 M KCl at 20 mV s^−1^ and, diverse scan rates (shown in the figure).

The voltammograms obtained on SCuNPs/SKPH in 0.005 M K_3_Fe(CN)_6_, 0.005 M K_4_Fe(CN)_6_, 0.1 M KCl redox at 20 mV s^−1^ and different scan rate is given in figure 7. It a show ΔEp is 0.1253 V at 20 mV s^−1^, this indicates that the electron transfer is slow due to the porosity of carbon. The EASA was determined using CV curves (Figure 7b) and the Randles‐Servik equation 2., ^[22]^

(2) $$I_{p}=2.69\times 10^{5}n^{3/2}AD^{1/2}Cv^{1/2}$$

Where Ip=redox (Ipa/Ipc) current, n=number electron transferred in the redox system (n=1), A=active surface area of electrode (cm^2^), D=diffusion coefficient (cm^2^ s^−1^), C=concentration of Fe(CN)_6_]^−4/−3^ solution (mol cm^−3^), and ν=scan rate (V s^−1^), respectively. The EASA was determined of 0.10 cm^2^. The surface area of polycrystalline Cu was 1 cm^2^.

### Study of the Electrodeposition of Copper on the CSKPH Film in an Acid Medium by Cyclic Voltammetry of Inversion Potentials

**Figure 8:**
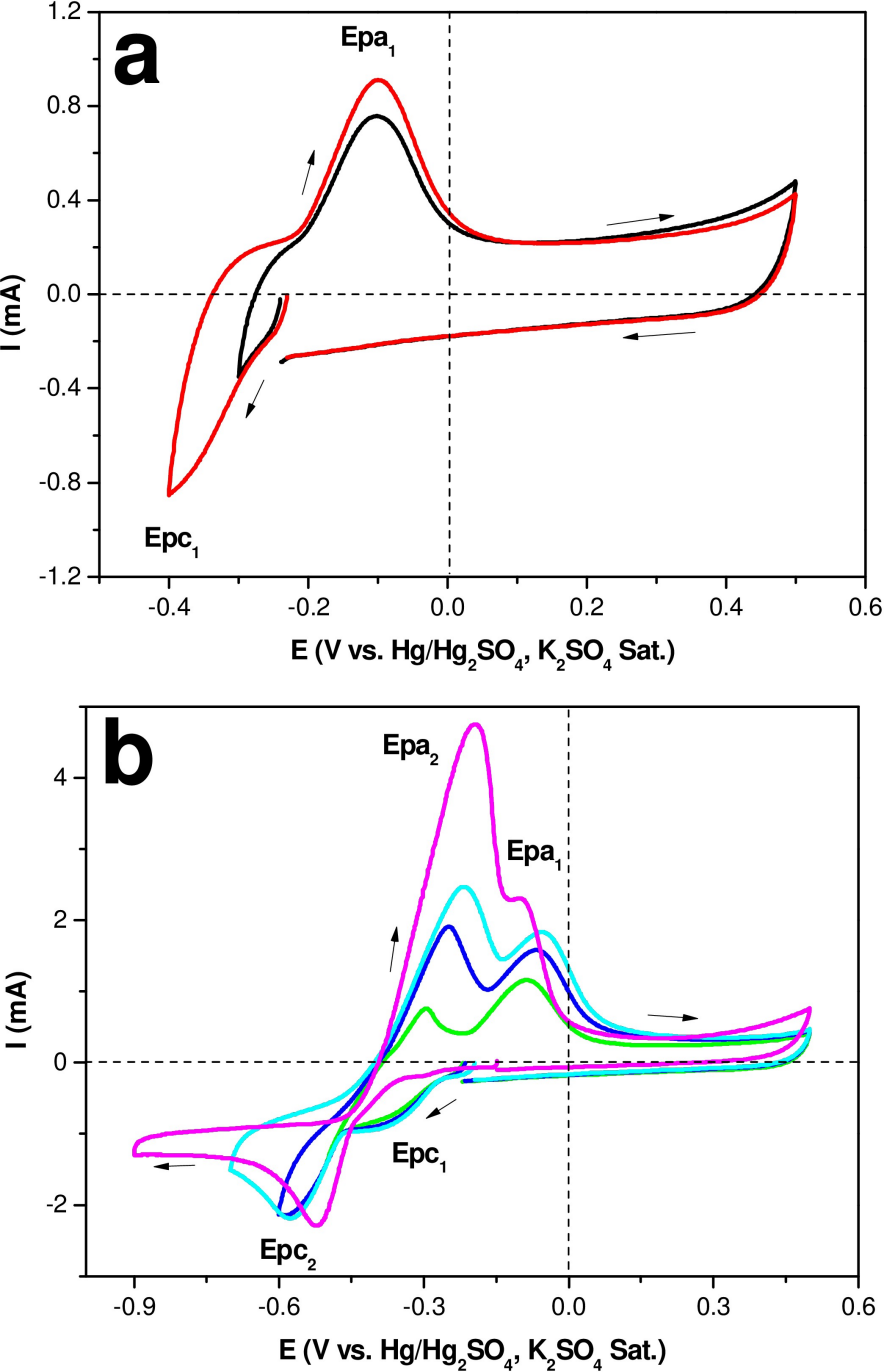
Voltammetric responses were obtained on the CSKPH carbon film in the 5x10^−2^ M CuSO_4_, 0.1 M K_2_SO_4_ system with pH=2.3 at 5 mV s^−1^, in the potential range between −0.9 V and 0.5 V.

Figure 8 shows the CV responses obtained by applying different potential limits (Eλ) at 100 mV intervals on the CSKPH film in the electrolyte 5×10^−2^ M CuSO_4_, 0.1 M K_2_SO_4_ at pH=2.3 to 5 mV s^−1^. In all cases, the scan was initiated in a negative direction regarding the zero current potential. When opening the potential window to more negative Eλ, the first cathodic peak (Epc_1_), associated with the reduction from Cu^2+^ to Cu^+^, is observed. When inverting the scan direction, the anodic peak (Epa_1_) associated with copper oxidizing is described (Figure 8a).

As the window opens to more negative potentials (Figure 8b), a second cathodic peak potential (Epc_2_) is described, related to the process of reduction from Cu^+^ to Cu and two processes associated with the oxidation of copper, Cu to Cu^+^ (equation 3) and Cu^+^ to Cu^2+^ (equation 4), represented for the following relations:

(3) $${Cu}^{2+}+e^{-}\to {Cu}^{+}$$

(4) $${Cu}^{+}+e^{-}\to Cu$$

As can be seen, copper reduction and oxidation processes are clearly described in the voltammetric response, corresponding to the range from −0.9 V to 0.5 V (pink line). The electrode morphology and the electrolytic medium are electrochemical variables that influence the stages of copper electrodeposition. Electrodeposition of copper in an acid medium in one stage using flat surfaces has been reported;[^23^, ^24^] in an alkaline medium using porous surfaces, two stages have been reported.^[25]^ This study shows the reduction of copper on activated carbon films from *Sargassum* spp. in an acid medium in two stages as a consequence the porous surface.

**Figure 9:**
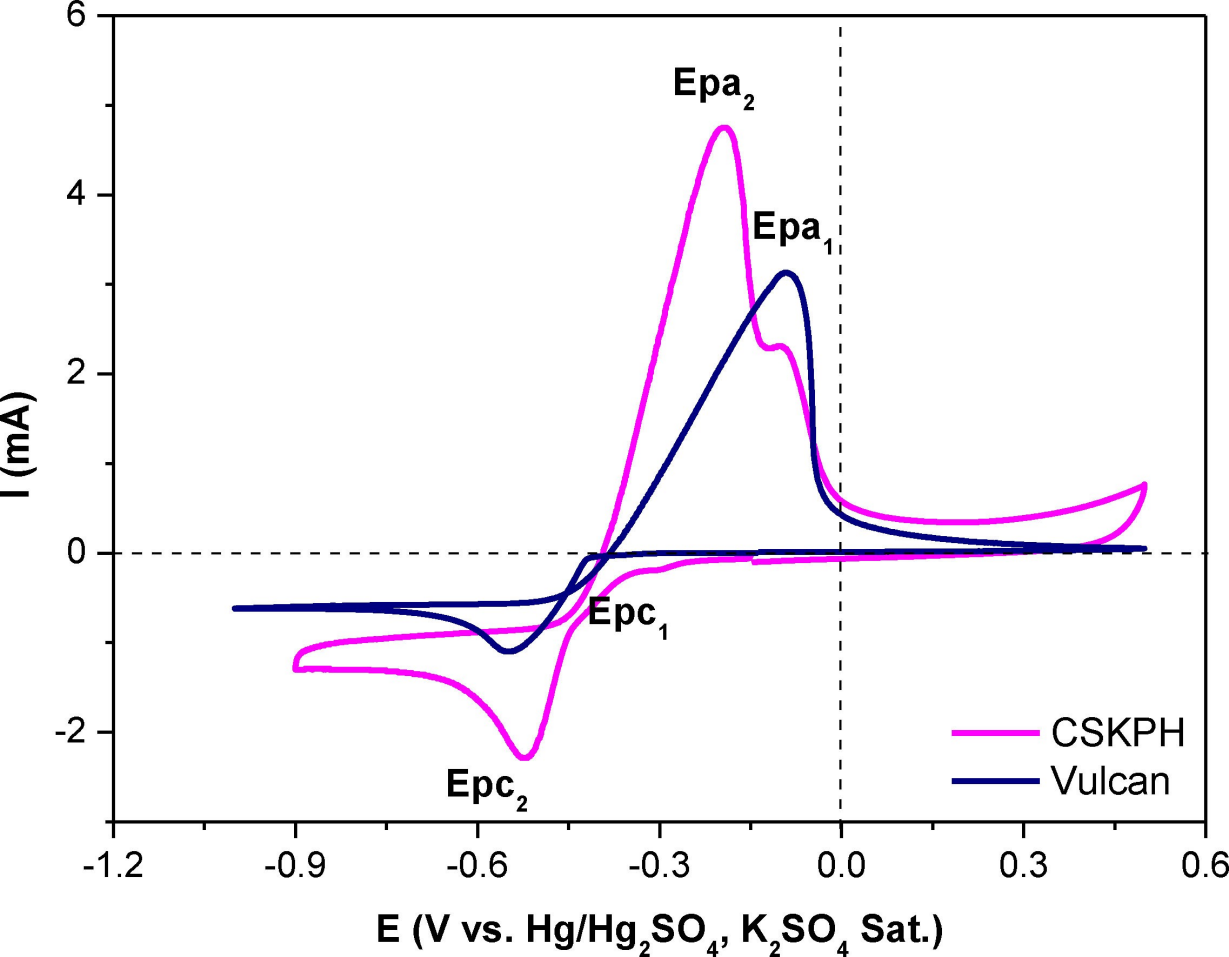
Voltammetric responses obtained on the carbon films (indicated in the figure) in the 5x10^−2^ M CuSO_4_, 0.1 M K_2_SO_4_ system with pH=2.3 in the potential range between 0.5 V to −1.0 V at 5 mV s^−1^.

In order to show that the porous surface influences the copper electrodeposition process in an acid medium, Figure 9 compares CSKPH film and Vulcan carbon. Although, in the Vulcan film, a reduction (Epc_1_) and an oxidation (Epa_1_) peak are described, this indicates that the copper electrodeposition is carried out in a single stage (equation 5). In contrast, in the CSKPH film, an Epc_1_ and two processes of oxidation associated with freshly electrodeposited copper are described. The differences may be related to the properties of the carbon, that is, the porosity since the Vulcan carbon has low porosity. At the same time, the CSKPH contains mesopores and micropores, where copper can be lodged.

(5) $${Cu}^{2+}+2e^{-}\to Cu$$

Table 4 shows the determined cathodic and anodic peak potential values based on the voltammetric responses displayed in Figure 9.

**Table 4:** Cathodic and anodic peak potential values obtained from the voltammetric responses of the carbon films in the 5×10^−2^ M CuSO_4_, 0.1 M K_2_SO_4_ system at pH=2.3 at 5 mV s^−1^.

Electrodes	Reduction		ΔE=Epa_1_^−^ Epc_1_	Oxidation	
	Epc_1_ (V)	Epc_2_ (V)		Epa_1_ (V)	Epa_2_ (V)
CSKPH film	−0.306	−0.519	0.209	−0.096	−0.196
Vulcan film	−0.095	–	0.45	−0.545	–

### Catalytic Activity of CuNPs/CSKPH in the Electrochemical Reduction of CO_2_

**Figure 10:**
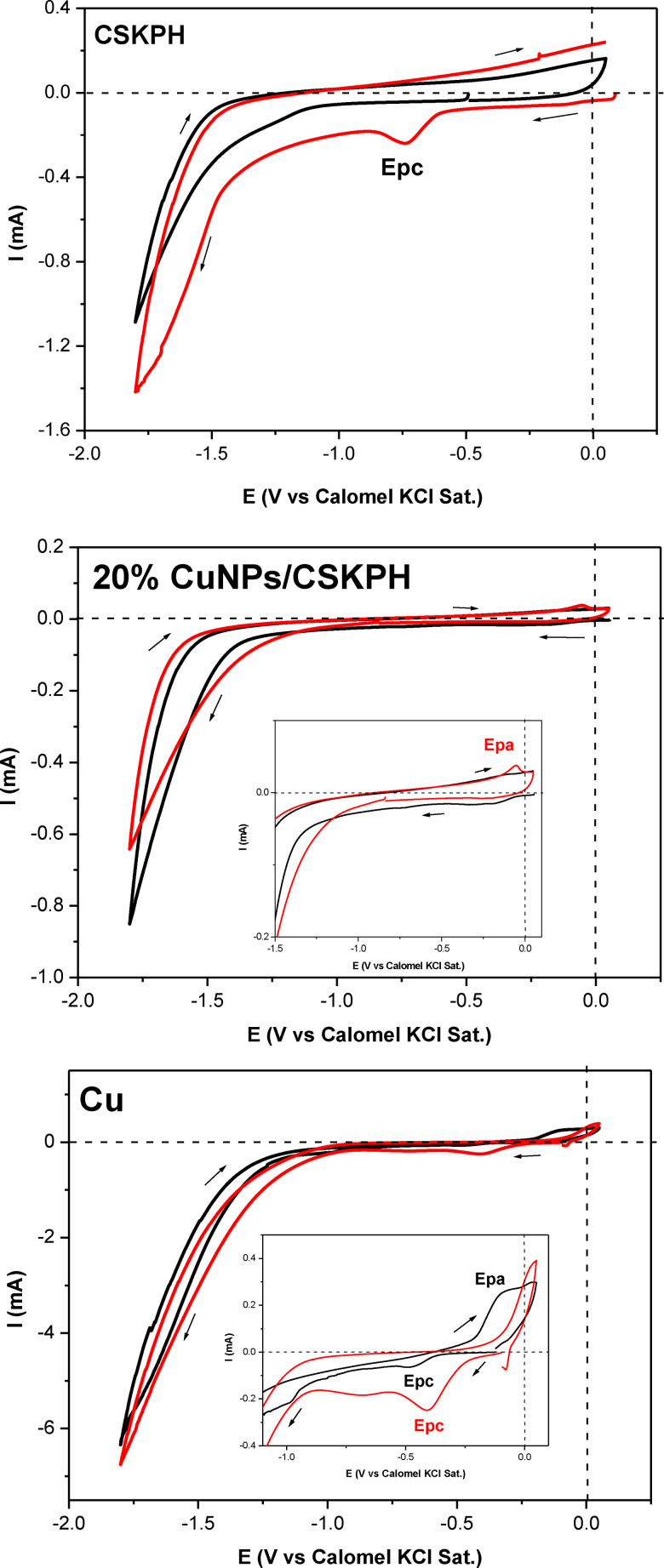
Comparison of the voltammetric response obtained on the CSKPH film, 20 % CuNPs/SKPH, and copper in the 0.1 M KHCO_3_ system in an N_2_ atmosphere with pH=8 (black line) and a CO_2_ atmosphere with pH=6.4 (red line) in the range from −1.8 V to 0.05 V to 5 mV s^−1^.

The catalytic activity of CuNPs/CSKPH was evaluated in the electrochemical reduction of CO_2_ by CV. Figure 10 compares the voltammetric responses obtained on the CSKPH film, 20 % CuNPs/CSKPH, and copper in the 0.1 M KHCO_3_ system with N_2_ and CO_2_ atmospheres at 5 mV s^−1^. In all cases, the scan started from zero current potential. Comparing the responses of the CSKPH film in both atmospheres, a cathodic peak potential (Epc=−0.73 V) associated with the surface chemistry of the coal and the current increase caused by the presence of CO_2_ is described.^[26]^ On the other hand, in the response of the 20 % CuNPs/CSKPH catalyst, an Epa of −0.052 V is observed to be associated with a copper oxide contained in the catalyst and a decrease in the cathodic current of −1.6 V, indicating the inhibition of the formation of hydrogen. In the response of copper, Epc and small shoulders associated with the redeposition of copper previously formed in the bicarbonate medium or copper oxides formed in the presence of oxygen are described.^[27]^

**Figure 11:**
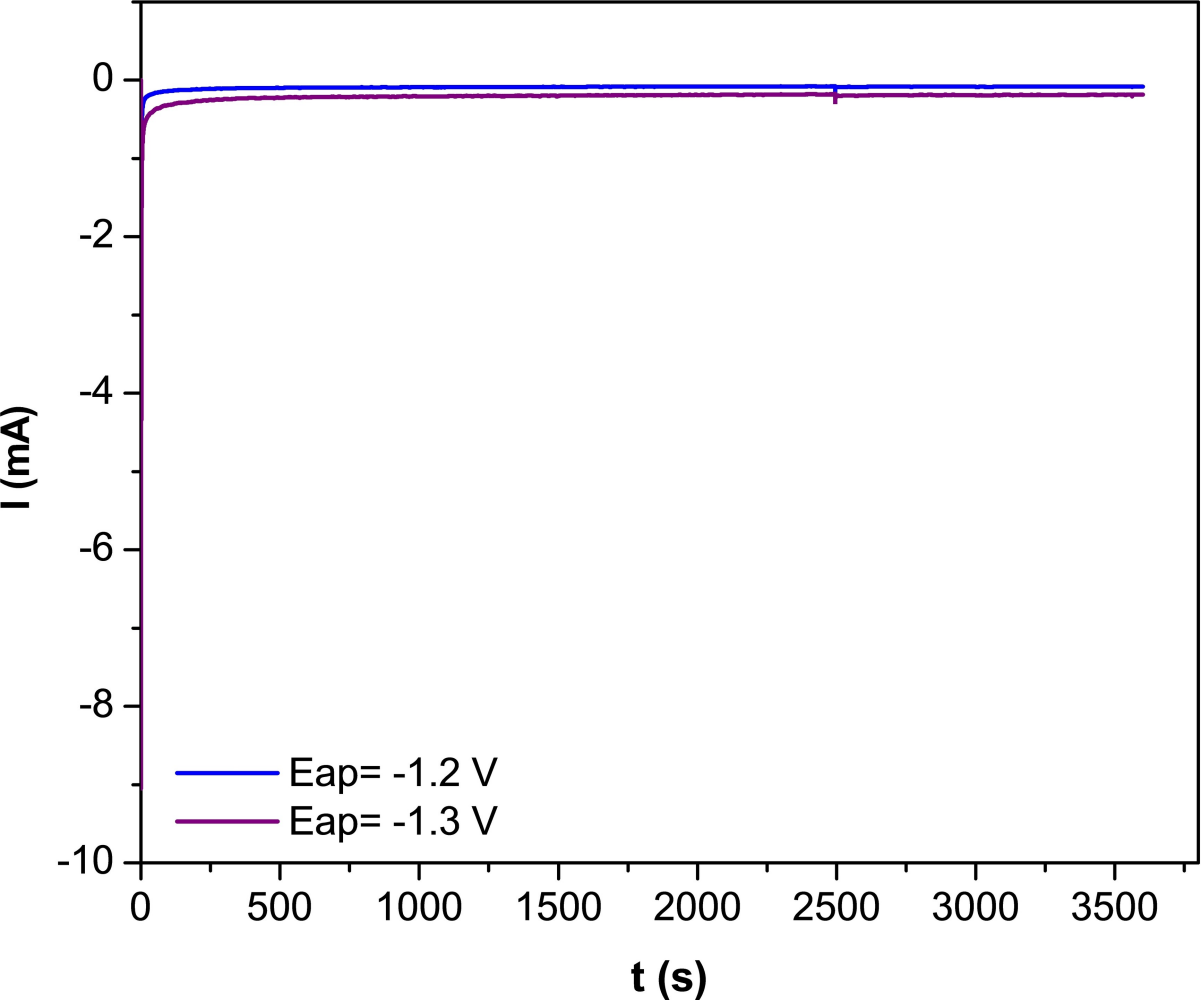
Current vs time obtained on 20 % ScuNPs/CSKPH in a CO_2_‐saturated 0.1 M KHCO_3_ solution applied potential different for 1 hour.

The stability of 20 % ScuNPs/CSKPH catalyst was investigated in a 0.1 M KHCO_3_ solution saturated with CO_2_ (pH=6.8) at an applied constant potential of −1.2 V and −1.3 V for 1 hour (see Figure 11). The electrochemical reduction current remains stable after 1 h. This indicates that the catalyst exhibits excellent stability toward the electrochemical reduction of CO_2_.

The performance of a catalyst is dependent on its faradaic efficiency for producing CO_2_ reduction products. Various methods such as precipitation, solvothermal, wet chemical, electrochemical, thermal treatment, plasma, sputtering, CVD, and electrospray have been reported for the preparation of Cu, CuO, Cu_2_O catalysts including nanocubes, nanodendrites, and monocrystals.[^28^, ^29^] Mayor products of C_2_H_4_ and C_2_H_6_O have been obtained in KHCO_3_ and KOH. However, there are few studies using copper or copper oxide supported on carbon (carbon Vulcan, carbon nanotubes), which have achieved a faradaic efficiency of 3 %(HCOO^−^), 13.4 % C_2_H_4_.[^30^, ^31^, ^32^]

Our study describes a green synthesis method that utilizes *Sargassum* spp as a raw material to produce a catalyst with a crystal size of 13 to 17 nm that accounts for 36 % by weight of the CSKPH (biochar). The morphology of the SCuNPs‐3 corresponds to nanoflakes formed by the accumulation of CuO and Cu nuclei, which aggregate to form flower‐like particles growing in different directions with almost uniform sizes. Although we did not quantify the CO_2_ products, we evaluated the catalyst‘s catalytic activity in the CO_2_ reaction and its stability for 1 hour.

## Conclusions

In summary, green synthesis of Cu and CuO nanoflowers using Sargassum spp. extract was fabricated on the CSKPH carbon film‘s surface and tested in the electrochemical CO_2_ reduction reaction. The crystalline nature of the Cu and CuO synthesized was confirmed by XRD analysis, obtaining an interplanar distance of 2.3 Å corresponding to CuO. TEM revealed the formation of particles in a nanoflower‐like structure. The voltammetric study allowed determine the concentration of copper present in the 20 % CuNPs/CSKPH catalyst. Electrodeposition of copper onto the CSKPH film is carried out in two stages due to the porosity of the carbon. The 20 % CuNPs/CSKPH catalyst showed catalytic activity in CO_2_ reduction and hydrogen inhibition.

In this way, it is possible to take advantage of the large quantities of *Sargassum* spp. in the Mexican Caribbean and offer an excellent alternative to use it as a material for synthesizing copper particles that, in turn, allow the reduction of CO_2_ emissions in the atmosphere. The use of these materials represents a positive impact on the environment.

## Experimental section

### Biochar Synthesis

*Sargassum sp*. was collected from the coast in Cancún, Quintana Roo; it was cleaned with seawater to remove sand and other impurities. Later, it was washed with tap and deionized water to clear out the remaining minerals and let dry in the sun for 72 hours. The obtained material was crushed in a PAGANI semi‐industrial grinder with a mesh of 1 mm and was sieved with a mesh number 100 to obtain fine sargassum dust. This material was pyrolyzed and activated with KOH following the workgroup methodology^[14]^ and the biochar was labeled as CSKPH.

### Green Synthesis of Copper Nanoparticles

The Sargassum Extract (SE) was prepared with 10 g of sargassum dust in 100 mL of deionized water; the solution was warmed until it reached the boiling point for 10 minutes. It was left to cool at room temperature and was filtered with Wathman No. 1 paper. Subsequently, three samples of Sargassum‐bases copper nanoparticles (SCuNPs) were synthesized at different proportions of SE and copper precursor (1 mM CuCl_2_.2H_2_O). First, copper nanoparticles were synthesized as described below: ScuNPs‐1 was obtained from 5 mL of SE and 95 mL of copper precursor, SCuNPs‐2 used 10 mL of SE and 190 mL of precursor, and finally, SCuNPs‐3 was prepared from 17 mL of SE and 400 mL of precursor. Each sample was shaken at 200 rpm and heated to 60 °C for 1 h. Afterward, they were heat treated in a Thermo Scientific oven at 400 °C, with a heating velocity of 10 °C min^−1^. Finally, the samples were washed with abundant deionized water. The quantity of coppe was different for each sample, 4 mg, 30 mg, and 37.1 mg for the samples SCuNPs‐1, SCuNPs‐2, and SCuNPs‐3, respectively. Considering the greater nanoparticle quantity obtained with the SCuNPs‐3 sample, this was supported on the biochar CSKPH to synthesize the electrocatalysts (SCuNPs/CSKPH).

### Green Synthesis of Catalyst SCuNPs/CSKPH

SCuNPs were supported on the biochar CSKPH varying the weight percentage. Table 5 summarizes the preparation of the samples by green synthesis; for example, 19 mg of CSKPH was mixed with 1 mg of the sample SCuNPs‐3 to obtain 5 wt % NPs. The interaction metal support is achieved by mixing both samples in 1.0 mL of ethanol (96 % purity) and homogenizing them in a sonic bath for 1 h to facilitate the SCuNPs dispersion. This sample was labeled as 5 % SCuNPs/CSKPH. Afterward, the samples were dried in an oven for 12 h at 70 °C, later characterized by XRD. The same procedure was performed for the 20 % SCuNPs/CSKPH sample.

**Table 5:** Relation of SCuNPs samples with CSKPH to obtain the catalysts.

Electrocatalyst	SCuNPs (mg)	Biocarbon CSKPH (mg)	Ratio (% mass SCuNPs)
5 % SCuNPs/CSKPH	1	19	5
20 % SCuNPs/CSKPH	4	16	20

### SCuNPs/CSKPH Films Preparation

Two catalytic inks were prepared, which contained 1 mg %SCuNPs/CSKPH, 3.1 mL of isopropyl alcohol, and 0.16 mL of liquid Nafion, which were submitted to ultrasonic shake for 30 minutes. The SCuPNs/CSKPH film electrode was obtained by placing 10 μL of ink over vitreous carbon (GC, geometric area 0.1963 cm^2^).

### Physical and Morphological Characterization

SCuNPs and SCuNPs/CSKPH samples were analyzed by X‐ray diffraction in the Bruker brand Phaser D2 equipment with Cu−Kα radiation (40 kV, 40 mA), the assessment conditions were from 10° to 100° incrementing 0.01° every 0.2 seconds. The surface morphology of the sample 20 % SCuNPs/CSKPH was characterized with a Hitachi S‐5500 Scanning Electron Microscope (SEM) at 5.0 kV and 40.0 k. Transmission Electron Microscopy was carried out using the TEM TALOS F200 equipment; the samples were analyzed in STEM mode, with an acceleration voltage of 200 kV.

### Electrochemical Characterization

The SCuNPs/CSKPH films were tested by cyclic voltammetry (CV) using a typical cell with three electrodes, consisting of a graphite rod as an auxiliary electrode, a saturated calomel electrode as a reference, and as a working electrode, the biochar films SKPH and SCuNPs/CSKPH. The electrolytic system of 0.1 M K_2_SO_4_ at pH=2.3 to 5 mV s^−1^ was used to identify the copper present in the catalyst in order to describe the electrodeposition of copper on the CSKPH film, the medium of 5×10^−2^ M CuSO_4_, 0.1 M K_2_SO_4_ at pH=2.3 to 5 mV s^−1^. The EASA of SCuNPs/CSKPH was determined by cyclic voltammetry in a 0.005 M K_3_Fe(CN)_6_, 0.005 M K_4_Fe(CN)_6_, 0.1 M KCl solution at different scan rates. Finally, the catalytic activity of the SCuNPs/CSKPH in the electrochemical reduction reaction of CO_2_ was characterized using the system 0.1 M KHCO_3_ in N_2_ and CO_2_ atmospheres. All the tests were carried out in the BioLogic® potentiostat/galvanostat, and the acquisition and processing of the voltammetry responses were obtained using the Ec‐Lab® V.10.38 software. Likewise, the experiments were carried out at room temperature and atmospheric pressure.

## Conflict of interests

The authors declare no conflict of interest.

1

## Data Availability

The data that support the findings of this study are available from the corresponding author upon reasonable request.
